# Paraneoplastic Dermatomyositis Preceding Gastroesophageal Junction Adenocarcinoma: A Case Emphasizing the Importance of Early Malignancy Screening

**DOI:** 10.7759/cureus.108425

**Published:** 2026-05-07

**Authors:** Minisha Kanakarajan McKinney, Oluwatomilola Oyasiji, Ndidi Enwereji, Meena Moossavi

**Affiliations:** 1 Department of Dermatology, Wayne State University School of Medicine, Detroit, USA; 2 Department of Dermatology, Wayne State University, Detroit, USA

**Keywords:** cancer-associated myositis, dermatomyositis, gastroesophageal junction adenocarcinoma, malignancy screening, paraneoplastic syndrome

## Abstract

Dermatomyositis (DM) is an idiopathic inflammatory myopathy marked by proximal muscle weakness and pathognomonic rashes such as heliotrope rash and Gottron papules. DM is associated with an increased risk of malignancy compared to the general population, with 15-30% of cases being paraneoplastic. DM-associated malignancies are often diagnosed at late stages, resulting in cancer being a leading cause of death in this condition. Additionally, DM can mask malignancy by presenting with similar symptoms as the inciting cancer, further emphasizing the importance of early, disease-specific cancer screening.

We report a 51-year-old woman with refractory DM presenting with muscle weakness, cutaneous lesions, and dysphagia. Lab tests showed elevated creatine kinase, positive ANA, and negative anti-Jo-1; muscle biopsy confirmed DM. Despite treatment for DM, her symptoms persisted. After complaints of worsening dysphagia, subsequent imaging, endoscopy, and biopsy revealed gastroesophageal junction (GEJ) adenocarcinoma. After receiving immunosuppressive treatment, the patient developed Pseudomonas pneumonia. Unable to recover, she rapidly declined and died in mid-April 2025.

This case emphasizes the need for early myositis-specific malignancy screening in new-onset DM, particularly when systemic or gastrointestinal symptoms are present. This will enable timely diagnosis and improve patient outcomes. Paraneoplastic DM often carries a more aggressive course, and while gastrointestinal (GI) cancers are highly associated with the disease, association with GEJ adenocarcinoma is rare. A high index of suspicion for malignancy should be maintained in new-onset DM, especially when systemic symptoms are present. Early malignancy screening tailored to myositis-associated malignancies at the time of diagnosis is critical to improve prognosis and facilitate timely intervention in this life-threatening paraneoplastic syndrome.

## Introduction

Dermatomyositis (DM) is a rare idiopathic inflammatory myopathy characterized by proximal muscle weakness and pathognomonic skin findings. Although most cases of DM are idiopathic, 15-30% are paraneoplastic and associated with underlying malignancy [1]. Adult DM patients face a 4.66-fold higher risk of malignancy compared to the general population, with the risk most pronounced within the first 12 months of DM diagnosis [2]. Unfortunately, DM-associated malignancies are often diagnosed at a late stage, contributing to cancer being a leading cause of death in adults with idiopathic inflammatory myopathy [3]. This could be due to conventional screening tools being ineffective at detecting malignancies early in DM patients, and current screening workflows largely mirroring those used in the general population rather than being tailored to DM-specific characteristics [4].

Current guidelines for malignancy screening in DM suggest screening be performed based on risk status, and the initial assessment should contain basic laboratory studies and imaging. In addition to malignancy screening, testing for common myositis-specific autoantibodies (MSAs) in patients with new-onset DM can identify MSAs associated with an increased cancer risk. This can play a role in guiding clinicians in their level of vigilance, particularly within the first year of DM onset. With gastrointestinal (GI) malignancies being one of the most common malignancies associated with DM, particularly pancreatic cancer, followed by stomach/gastric cancer and colorectal cancer, early malignancy screening should be highly considered in patients with new-onset DM and dysphagia [2]. In addition to GI cancers, the most frequently associated malignancies include cancers of the lung, cervix, ovary, pancreas, bladder, as well as non-Hodgkin lymphoma [2].

We present a case of DM in which gastroesophageal junction (GEJ) adenocarcinoma was identified after progressive constitutional and gastrointestinal symptoms, highlighting the critical role of early, DM-specific malignancy screening for improved cancer detection rates and patient outcomes.

## Case presentation

A 51-year-old woman with no significant past medical history presented to an outside hospital in December 2024 with a 3-month history of progressive proximal muscle weakness, facial swelling, fatigue, and characteristic cutaneous findings, including a heliotrope rash and Gottron papules. Laboratory testing at that time revealed significantly elevated creatine kinase, concerning for myopathy. Antibody testing was performed and showed a positive antinuclear antibody (ANA), while anti-SSA/SSB and anti-Jo1 were negative. A muscle biopsy was subsequently performed and revealed inflammatory myopathy, supporting the diagnosis of dermatomyositis (DM). She was initiated on high-dose corticosteroids 80 mg, which was tapered to 10 mg, diphenhydramine 25 mg, intravenous immunoglobulin (IVIG) every 4 weeks, and methotrexate (MTX) 15 mg weekly in December 2024 for the treatment of DM. Concurrent symptoms of weight loss, early satiety, and intermittent dysphagia were initially attributed to DM-associated myopathy.

**Figure 1:**
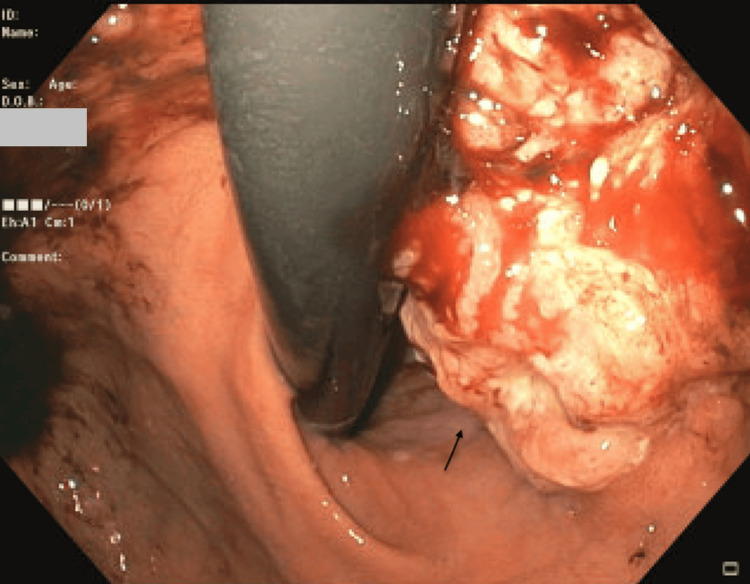
Esophagogastroduodenoscopy (EGD) displaying a gastroesophageal junction (GEJ) mass with retroperitoneal lymphadenopathy This image displays a mass to the right of the endoscopic probe, indicated by the black arrow.

In January 2025, she was hospitalized for another DM flare and reported worsening dysphagia and decreased appetite despite adherence to treatment for her DM, raising concern for an alternate cause for her symptoms. Computed tomography (CT) of the abdomen was performed and revealed a GEJ mass with retroperitoneal lymphadenopathy, shown in Figure 1. Gastroenterology was consulted, and endoscopic ultrasound with biopsy performed during hospitalization confirmed poorly differentiated GEJ adenocarcinoma, stage T3N1MX. She underwent percutaneous endoscopic gastrostomy (PEG) tube placement and began FOLFOX (folinic acid, fluorouracil, and oxaliplatin) chemotherapy in the third week of January 2025. By mid-February 2025, she was discharged from inpatient rehabilitation with improved muscle strength. She received her third cycle of FOLFOX in the first week of March 2025, with plans for additional cycles in the coming months. Methotrexate was being administered concomitantly with chemotherapy and was well-tolerated.

In March 2025, she presented to the emergency department with signs suggestive of a dermatomyositis flare, including periorbital edema with a violaceous hue consistent with a heliotrope rash, faint poikilodermatous erythema over the chest and upper back, dilated nailfold capillary loops, and periungual erythema. She also exhibited severe proximal muscle weakness and bilateral lower extremity edema. Laboratory evaluation revealed elevated creatine kinase, aspartate aminotransferase, alanine aminotransferase, antinuclear antibody, lactate dehydrogenase, and low albumin, as shown in Table 1. These laboratory results reflect the inflammation and muscle damage seen in dermatomyositis. Elevated aspartate aminotransferase (AST) and alanine aminotransferase (ALT) are possibly related to a combination of the release of small amounts of these enzymes from muscle cells during muscle damage and liver damage. During hospitalization, consults to hematology-oncology, gastroenterology, rheumatology, and dermatology were placed. Given concerns about immunosuppression during active chemotherapy, methotrexate was discontinued mid-March. Later that month, she re-presented with acute respiratory distress. Chest imaging showed bilateral pulmonary infiltrates and effusions. 

**Table 1:** Patient's significant laboratory values from mid-March 2025 The lab results correlate with inflammation and muscle damage seen in dermatomyositis.

Laboratory Test	Patient Value	Reference Range	Units
Creatine kinase (CK)	381	20-200	U/L
Aspartate aminotransferase (AST)	146	8-43	U/L
Alanine aminotransferase (ALT)	87	7–56	U/L
Lactate dehydrogenase (LDH)	661	125-225	U/L
Antinuclear antibody (ANA)	1:320	<1:80	Titer
Albumin	2.9	3.5–5.0	g/dL

During her hospitalization in March 2025, she developed septic shock and was admitted to the intensive care unit, requiring mechanical ventilation and vasopressor support. Sputum culture at the end of March 2025 grew *Pseudomonas aeruginosa*, leading to a diagnosis of pneumonia secondary to *Pseudomonas*. Despite aggressive management, she succumbed to complications related to her infection and died in mid-April 2025.

## Discussion

Cancer occurs in approximately 9-32% of adult DM patients, with a 3- to 4.66-fold increased risk compared to the general population [5]. As can be seen in Table 2, common associated cancers include those of the lung, ovary, cervix, bladder, GI tract, and lymphoid tissues [2]. There is an elevated risk of cancer in the three years before and after DM onset, defining a critical 6-year surveillance window. GI cancers are strongly represented in paraneoplastic DM cases, with colon (5.2-14%) and stomach (1-6%) cancers being commonly reported, as outlined in Table 2. The association of DM with GEJ adenocarcinoma, as seen in the patient in this case, is much less represented, leading to a potential discrepancy in correlating DM with GEJ adenocarcinoma.

**Table 2:** Malignant diseases with the highest incidence detected in patients with dermatomyositis * Asian population Source: [2]

Types of malignant diseases detected with the highest incidence in dermatomyositis	Percentage of certain types of malignant diseases in all patients with dermatomyositis and malignancy
Lymphatic and hematopoietic	3.6-17%
Lung	8-34%
Ovary	2-11.3%
Prostate	1-9.4%
Colon	5.2-14%
Breast	2.8-24.5%
Stomach	1-6%
Nasopharynx	22.5-62.5%*

The pathogenesis of cancer-related DM is best understood as a cross-reactive anti-tumor immune response. This means that as the body mounts a response against tumor cells, it mistakenly attacks the body’s normal structures, in this case, muscles and skin tissue, that share antigenic targets with the tumor [6]. The Neoantigen/Molecular Mimicry Model outlines several interconnected steps that describe DM as a paraneoplastic disease. Notably, somatic mutations in genes encoding myositis autoantigens, like TRIM33 (TIF1g), TRIM66 (NXP2), and MORC, within the tumor lead to highly immunogenic antigens that trigger an immune response [7]. Other mechanisms include clonal expansion of T cells, anti-TIF1g autoantibodies, and immune escape as causes for paraneoplastic DM, underscoring the complexity of diagnosing this condition.

Physicians must maintain a high index of suspicion for occult malignancy in patients with new-onset DM, especially when systemic symptoms, such as weight loss, dysphagia, or night sweats, are present. A detailed history and review of systems are critical to guide appropriate and individualized malignancy screening. Current guidelines state that cancer screening for DM patients should be performed based on their risk status. Additionally, studies have shown that certain myositis-specific autoantibodies (MSAs) can stratify the cancer risk. Anti-TIF1-γ has the strongest association with a 9.37-fold increased cancer risk. Anti-NXP2 was found to have a 3.68-fold increase in cancer risk. Anti-Jo-1, Anti-MDA5, and Anti-HMGCR were not associated with an increase in cancer risk [2]. Skin biopsy can aid in the diagnosis, with histopathologic findings including vacuolar interface dermatitis, mucin deposition, and superficial perivascular infiltrates [8]. A patient’s myositis subtype, autoantibody status, and clinical features, with DM, age >40 years, anti-TIF1γ or anti-NXP2 positivity, persistent high disease activity, dysphagia, and cutaneous necrosis are factors that identify whether they are at increased risk. At a minimum, initial assessment should include basic investigations, such as laboratory studies and chest imaging, while higher-risk patients may warrant more extensive screening with cross-sectional imaging (e.g., CT chest/abdomen/pelvis), targeted studies, such as mammography, pelvic imaging, or tumor markers, and consideration of positron emission tomography (PET)-CT or endoscopic evaluation if suspicion remains high. Physicians must balance diagnostic yield with potential procedural risks, utilizing shared decision-making to tailor evaluation based on clinical context and patient-specific risk factors [9].

## Conclusions

Dermatomyositis may serve as a harbinger of underlying malignancy, especially within the first year after diagnosis. Comprehensive and timely malignancy screening, including history, physical examination, and targeted investigations, allows for recognition of atypical or paraneoplastic features and earlier intervention. Myositis-specific autoantibodies can potentially provide important prognostic insight, as antibodies such as anti-TIF1γ and anti-NXP2 are associated with a higher likelihood of malignancy, whereas others, including anti-Jo-1, are linked to lower risk. This case highlights the rare association of dermatomyositis with gastroesophageal junction adenocarcinoma, underscoring the aggressive nature of paraneoplastic DM and the need for clinical vigilance and timely oncologic evaluation.
